# Radial extracorporeal shockwave promotes subchondral bone stem/progenitor cell self-renewal by activating YAP/TAZ and facilitates cartilage repair in vivo

**DOI:** 10.1186/s13287-020-02076-w

**Published:** 2021-01-07

**Authors:** Zhidong Zhao, Yuxing Wang, Qian Wang, Jiawu Liang, Wei Hu, Sen Zhao, Peilin Li, Heng Zhu, Zhongli Li

**Affiliations:** 1 Chinese People’s Liberation Army (PLA) General Hospital, Chinese PLA Medical School, No. 28 Fuxing Road, Haidian District, Beijing, 100853 China; 2 Beijing Institute of Radiation Medicine, No. 27 Taiping Road, Haidian District, Beijing, 100850 China; 3 Graduate School of Anhui Medical University, No. 81 Meishan Road, Shu Shan District, Hefei, 230032 Anhui Province China

**Keywords:** Extracorporeal shockwave, Stem/progenitor cells, Self-renewal, Cartilage regeneration, YAP/TAZ

## Abstract

**Background:**

Radial extracorporeal shockwave (r-ESW), an innovative and noninvasive technique, is gaining increasing attention in regenerative medicine due to its mechanobiological effects. Subchondral bone stem/progenitor cells (SCB-SPCs), originating from the pivotal zone of the osteochondral unit, have been shown to have multipotency and self-renewal properties. However, thus far, little information is available regarding the influences of r-ESW on the biological properties of SCB-SPCs and their therapeutic effects in tissue regeneration.

**Methods:**

SCB-SPCs were isolated from human knee plateau osteochondral specimens and treated with gradient doses of r-ESW in a suspension stimulation system. The optimized parameters for SCB-SPC self-renewal were screened out by colony-forming unit fibroblast assay (CFU-F). Then, the effects of r-ESW on the proliferation, apoptosis, and multipotency of SCB-SPCs were evaluated. Moreover, the repair efficiency of radial shockwave-preconditioned SCB-SPCs was evaluated in vivo via an osteochondral defect model. Potential mechanisms were explored by western blotting, confocal laser scanning, and high-throughput sequencing.

**Results:**

The CFU-F data indicate that r-ESW could augment the self-renewal of SCB-SPCs in a dose-dependent manner. The CCK-8 and flow cytometry results showed that the optimized shockwave markedly promoted SCB-SPC proliferation but had no significant influence on cell apoptosis. Radial shockwave exerted no significant influence on osteogenic capacity but strongly suppressed adipogenic ability in the current study. For chondrogenic potentiality, the treated SCB-SPCs were mildly enhanced, while the change was not significant. Importantly, the macroscopic scores and further histological analysis strongly demonstrated that the in vivo therapeutic effects of SCB-SPCs were markedly improved post r-ESW treatment. Further analysis showed that the cartilage-related markers collagen II and proteoglycan were expressed at higher levels compared to their counterpart group. Mechanistic studies suggested that r-ESW treatment strongly increased the expression of YAP and promoted YAP nuclear translocation in SCB-SPCs. More importantly, self-renewal was partially blocked by the YAP-specific inhibitor verteporfin. Moreover, the high-throughput sequencing data indicated that other self-renewal-associated pathways may also be involved in this process.

**Conclusion:**

We found that r-ESW is capable of promoting the self-renewal of SCB-SPCs in vitro by targeting YAP activity and strengthening its repair efficiency in vivo, indicating promising application prospects.

## Introduction

Radial extracorporeal shockwave (r-ESW), an innovative and noninvasive therapy technique, has been widely used in treating various musculoskeletal diseases [1–3]. More encouragingly, as a specific mechanical stimulus form, r-ESW has gained increasing attention in regenerative medicine due to its mechanobiological effects on stem cells [4–6]. Nevertheless, the molecular mechanisms of r-ESW remain largely indistinct [7, 8].

Subchondral bone [9], consisting of subchondral bone plate and cancellous bone, has been demonstrated to play a vital role in the pathogenesis of related diseases, such as cartilage/osteochondral injury, osteoarthritis, and osteochondritis dissecans [10–12]. It has become a potential therapeutic target of various therapeutic methods [13, 14]. However, as one predilection site for clinical diseases, including traumatic injury, inflammation, or even tumors, the mechanisms of injury and repair in subchondral bone are extremely complicated.

With the rapid development of regenerative medicine, an increasing number of endogenous stem/progenitor cells, including synovium-derived MSCs, synovial fluid-derived MSCs, intraarticular fat pad-derived stem cells, and cartilage-derived stem/progenitor cells, have been identified, applied, and targeted to repair cartilage injury [15–17]. However, few studies are available concerning the repair potential of stem/progenitor cells originating from the native subchondral bone region [18]. Previous studies have shown the multipotency and self-renewal properties of SCB-SPCs [19, 20]. Our previous study suggested that SCB-SPCs displayed superior self-renewal capacity than their bone marrow counterparts in rabbits [21]. Lian et al. [22] suggested that SCB-SPCs showed increased expression of stem cell makers and stronger proliferation capacity. Hence, the repair role of SCB-SPCs closely involved in osteochondral regeneration must be explored. In addition, we previously repaired cartilage injury by combining microfracture with extracorporeal shockwave and achieved remarkable improvement, but the mechanisms were still unknown [23]. Moreover, our recent work targeting bone marrow mesenchymal stem cells suggests that radial shockwave may promote its therapeutic effects [4]. Therefore, based on our previous findings and the literature, along with the pivotal role of SCB-SPCs, we hypothesize that r-ESW may enhance the properties of SCB-SPCs and that primed SCB-SPCs may yield more promising repair results.

In the current study, we harvested SCB-SPCs from human knee osteochondral specimens. Then, the SCB-SPCs were treated with gradient doses of r-ESW, and the cells from the optimized group were transplanted into the osteochondral injury model via the cell-PLGA construct in vivo. In addition, mechanosensitive YAP signals were selected to explore the underlying mechanisms that control the therapeutic effect.

## Methods and materials

### Ethics

The human knee plateau osteochondral samples used in this study were approved by the institutional ethical review board of the Chinese People’s Liberation Army General Hospital (rapid review and approval of scientific research projects for use of discarded biological materials), and informed consent was obtained from all donors.

### Isolation, cultivation, and characterization of stem/progenitor cells from human knee tibial plateau subchondral bone

Human SCB-SPCs were isolated from the subchondral bone of the knee lateral tibial plateau (K-L, 1-2 level). All osteochondral specimens used in our study were collected from patients diagnosed with primary knee osteoarthritis who received total knee arthroplasty (Table S1). After the osteochondral specimen was rinsed with 0.1 mol/L phosphate-buffered saline (PBS), the subchondral bone was cut into small fragments (2 mm × 2 mm × 2 mm) and digested for 2 h at 37 °C using 0.1% collagenase II (Sigma). Then, the bone chips were neutralized with alpha-minimal essential medium (α-MEM) supplemented with 10% fetal bovine serum (FBS) (Invitrogen Life Technologies), centrifuged at 500*g* for 10 min at 4 °C, and washed twice with PBS successively to obliterate residual tissue, after which the fragments were placed in 25 cm^2^ cell culture flasks containing α-MEM supplemented with 10% FBS and 100 U/ml penicillin and incubated at 37 °C in an atmosphere of 5% CO_2_. Medium exchange was performed every 3 days. After approximately 7–10 days, migrated cells outgrown from the subchondral bone chips could be observed. When the cells reached approximately 80–90% confluence, they were subcultivated using trypsin (0.25%, Invitrogen) and replated in 75 cm^2^ cell culture flasks. SCB-SPCs at passages 3–5 were used for the subsequent experiments unless otherwise stated.

Flow cytometry and trilineage induction were performed according to previous protocols [24]. Briefly, SCB-SPCs were suspended in PBS containing 5% bovine serum albumin (Sigma Aldrich, USA) at a concentration of 5 × 10^5^ cells/1 mL and incubated with phycoerythrin (PE)-conjugated monoclonal antibodies against human CD29, CD44, CD73, CD166, fluorescein isothiocyanate (FITC)-conjugated monoclonal antibodies against human CD45, CD90, CD271, and allophycocyanin (APC)-conjugated antibodies against CD31 and CD105 at 4 °C for 1 h. All antibodies were purchased from eBio-Science. Then, the cells were washed three times using PBS and incubated with fluorescein isothiocyanate (FITC)-labeled secondary anti-mouse antibody (1:200 dilution, Invitrogen) at 4 °C for 30 min. The appropriate rabbit isotype antibodies were used as controls. Samples were processed using a FACS Canto II flow cytometer (BD Biosciences, USA) and analyzed with FlowJo 7.6.

To verify the multipotency of SCB-SPCs, trilineage induction experiments were conducted. For osteogenic differentiation, passage 3 SCB-SPCs were cultured in 48-well plates at a density of 3 × 10^3^ cells/well supplemented with osteogenic induction medium (Cyagen, HUXMA-90021). For chondrogenic differentiation, 4 × 10^5^ SCB-SPCs were harvested in a 15-ml centrifuge tube followed by centrifugation to form the microsphere. Then, the pellets were cultured in chondrogenic induction medium (Cyagen, HUXMA-9004) for approximately 4 weeks. For adipogenic differentiation, passage 3 SCB-SPCs were cultivated in 48-well plates at a density of 5 × 10^4^ cells/well supplemented with adipogenic induction medium (Cyagen, HUXMA-90031). The induction medium was replaced every 3 days for approximately 2–3 weeks. After induction, the cells were processed for histological staining and quantitative real-time PCR (RT-PCR) analysis.

After osteogenic differentiation, the cells were stained with an ALP assay kit (Sigma-Aldrich) at 2 weeks and a Von Kossa kit (Sigma-Aldrich) at 4 weeks. After adipogenic differentiation, the cells were stained with Oil Red O (Sigma-Aldrich, USA) for 30 min to investigate the intracellular accumulation of adipocyte lipids. After chondrogenic differentiation, the microspheres were fixed with 4% formaldehyde and then paraffinized, sectioned and stained with HE, safranin O, toluidine blue, and immunohistochemical staining of Sox-9 and collagen II.

### Preparation of SCB-SPCs and direct r-ESW stimulation in a suspension system

To reduce the influence of transmission media such as cushions or gels in the process of energy transmission as much as possible, a floating culture system was used. Briefly, 4 × 10^6^ SCB-SPCs were harvested and resuspended in 20 ml of culture medium in 100-mm cell culture dishes. The shockwave applicator was maintained just below the surface of the liquid level to treat the distributing SCB-SPCs. Radial shockwaves were generated by a Swiss DolorClast Master (Electro Medical Systems SA, Switzerland). The treatment parameters applied were based on previous protocols and our preliminary experiments: continuous pulse, frequency 5 Hz, with 300, 600, 900, 1200 impulses, combined with 1, 2, and 3 bar; 0 bar served as the control group. Corresponding to the numbers of stimulations, the durations of r-ESW stimulation were 1, 2, 3, and 4 min, respectively. The optimized parameters selected by CFU-F were used for the following in vitro and in vivo experiments.

### Colony-forming unit fibroblast formation assay (CFU-F)

Passage 3 SCB-SPCs were trypsinized, resuspended, harvested, and prepared in 100-mm culture dishes. After gradient doses of shockwave treatment, aliquots of cell suspensions were allocated to six-well culture plates at a concentration of 2 × 10^3^ cells/well and cultured for approximately 10 days (three replications per group). Crystal violet was used to stain the colonies, and the number was recorded and analyzed using microscopic investigation (≥ 50 cells). Gross appearances were imaged vertically by digital photography. Based on the comprehensive analysis results of the colony, an optimized energy parameter was chosen for the subsequent experimental studies.

### The effects of optimized r-ESW on SCB-SPC proliferation, apoptosis, and trilineage differentiation

The Cell Counting Kit-8 (CCK8, Dojindo) was applied to investigate the influence of radial shockwave on SCB-SPC proliferation. SCB-SPCs were seeded at a density of 2 × 10^3^ cells/well (five wells in each group) in 96-well plates and then measured at individual points in time. Ten microliters of reagents were added to each well, followed by incubation at 37 °C for 1 h. Then, the absorbance value at 450 mm was measured by a microplate reader.

Apoptosis tests were performed according to the manufacturer’s instructions. Briefly, 1 × 10^5^ cells/sample were prepared in 100 μl of binding buffer in a labeled tube. Then, Annexin V and propidium iodide (PI) (Sigma-Aldrich, 5 μl each) were successively added to the tube with vortexing and incubation. PBS was then added and gently vortexed before the samples were analyzed by flow cytometry.

To further investigate the effect of radial shockwave on the differentiation ability of SCB-SPCs, we subjected SCB-SPCs to r-ESW stimulation prior to multidifferentiation induction.

### Real-time quantitative PCR analysis (RT-PCR)

Total RNA was extracted by using TRIzol reagent (Invitrogen, USA) from both r-ESW-treated and untreated SCB-SPCs. The RNA was then reverse-transcribed into cDNA by a DNA synthesis kit (TaKaRa, Shiga, Japan). When assessing gene changes related to differentiation and self-renewal, the SCB-SPCs were cultured in osteogenic, adipogenic, and chondrogenic induction medium for 7 days before they were harvested. Human Nanog, Sox-2, Runx-2, OCN, CEBPα, PPARγ, Sox-9, and collagen type II (Col-II) cDNAs were amplified by real-time PCR using a SYBR PCR Master Mix Kit (Sigma-Aldrich). Real-time PCR was performed with a real-time PCR detection system (ABI, Foster City, USA). The 2^−ΔΔCT^ method was used to analyze the relative gene expression levels. The primer sequences used in this study are listed in the Table S2.

### Western blot assay

On the basis of the CFU-F results, several gradient parameters were selected (0 bar as the control; 1 bar 300 times; 2 bar 600 times; and 3 bar 1200 times as the experimental groups) to explore the possible mechanisms of r-ESW. Passage 3 SCB-SPCs were cultured in a 75 cm^2^ culture flask and starved in serum-free α-MEM medium for at least 12 h before radial shockwave stimulation. After shockwave treatment with the aforementioned energy parameters, the protein samples of SCB-SPCs were collected using protein lysis buffer (Bio-Rad, Hercules, CA, USA). The extracted cellular proteins were separated by 10% SDS-PAGE-denaturing gels. After electrophoresis, proteins were transferred/electroblotted onto a polyvinylidene difluoride membrane and blocked in 5% wt/vol nonfat dry milk. The membranes were incubated with anti-YAP, anti-TAZ, and anti-GAPDH (CST, USA) antibodies at 4 °C overnight. After washing three times using Tris-buffered saline containing Tween-20 (TBST), horseradish peroxidase (HRP) secondary antibody was diluted to 1:1000 in 5% nonfat dry milk in TBST solution and then incubated with the membrane at room temperature for 1 h. Residual secondary antibody was rinsed off from the membrane with TBST, and a chemiluminescent signal was generated by using the detecting reagents (ECL western blotting Substrate, 32,106, Thermo Scientific, USA) according to the manufacturer’s protocols. The western blotting assay was performed at least 3 times independently, and representative results are shown below. The intensities of the immunoreactive proteins were measured by computerized image analysis and normalized to GAPDH levels. Experiments were repeated at least 3 times.

### Immunofluorescence staining

According to the intervention factor, the control, r-ESW and r-ESW + verteporfin groups were set. The verteporfin concentration was screened out by safety evaluation (Fig. S1). A total of 4 × 10^4^ SCB-SPCs were cultured on glass coverslips in 12-well plates in every group (*N* = 5). After approximately 24 h of cultivation, cells were fixed at room temperature with 4% paraformaldehyde for 20 min followed by permeabilization with 0.2% Triton X-100 for 15 min. After washing with PBS 3 times, samples were incubated with 1% BSA-PBS for 30 min to increase specific binding. Then, the samples were incubated overnight with primary antibodies against YAP (CST), Nanog (Invitrogen), and Sox2 (Abcam) at 4 °C. Samples were rewarmed and incubated with secondary antibodies at room temperature for 1 h. Then, DAPI was used to stain the nuclei. After dyeing was completed, the samples were washed with PBS, and the images were captured by laser confocal microscopy (Leica TCS SP8 Scan).

### YAP localization qualification

Referring to previous studies [25], the relative localization proportion of YAP was evaluated with the following status: prior nuclear localization (N), indicating that YAP almost overlapped with the nucleus; homogeneous distribution (N/C), indicating that the protein was located in both the cytoplasm and nucleus; and prior cytoplasmic distribution, indicating that the protein was mainly localized in the cytoplasm and barely observed in the nucleus (C). Percentages were used to present the data. Values are means ± SD (*n* = 3).

### The in vivo repair efficiency of SCB-SPCs pretreated by optimized radial shockwave

#### Animals

Fifty healthy New Zealand white rabbits aged 120 days with body weights ranging from 2.5 to 3 kg were incorporated into this 6- and 12-week study. Rabbits were kept in a standardized feeding environment. Animals were supplied by the Experimental Animals Center of the Chinese People’s Liberation Army (PLA) General Hospital. Experimental protocols in our operation procedure were in compliance with the Animal Welfare Act and were approved by the Animal Care and Use Committee of the Laboratory Animal Research Center at the PLA General Hospital (Reference number: 2019-X15-57).

To evaluate the in vivo repair efficiency of SCB-SPCs pretreated by optimized shockwave stimulation, polylactic-co-glycolic acid (PLGA) was prepared by hole puncher (4.5 mm in diameter and 4 mm in thickness) and was used as cell carrier scaffolds following our previous protocol [26]. The constructs were then implanted into the osteochondral defects model in rabbits. Shockwave-treated SCB-SPCs were seeded onto the prepared PLGA films and cultured in medium for 12 h. The osteochondral defect model of the rabbit knee was implemented following our previous protocol. In brief, anesthesia was administered by peritoneal injection of ketamine/xylazine/buprenorphine. A medial parapatellar incision was created after shaving and depilating, and then the patella was everted for sufficient exposure of the trochlear femur and distal femur. A cylindrical osteochondral defect of 4.5 mm in diameter and 5 mm in depth was created in the trochlear femur using a sterile trephine. Then, the models were treated with the respective interventions. All rabbits were randomly allocated into 5 different groups at 6 and 12 weeks. Group A, osteochondral defects without treatment (control group). Group B, defects with PLGA scaffolds only. Group C, defects with constructs composed of unprocessed SCB-SPCs and PLGA scaffolds. Group D, defects with constructs composed of radial-shockwave treated SCB-SPCs and PLGA scaffolds and Sham group (Table S3). Among the two latter groups, 1 × 10^6^ SCB-SPCs were incorporated onto the PLGA films before they were implanted into osteochondral defects. At the end of the surgery, the patella was returned to its original position, and then the capsule and skin were closed using resorbable sutures and nylon wire, respectively. All animals were allowed to move freely in cages after anesthesia. Intramuscular penicillin injections were given to each rabbit to prevent infection.

#### Pathological analysis of repaired tissues

At 6 and 12 weeks after the operation, the animals were euthanized for investigations. The trochlear femur was harvested and photographed by a digital camera for macroscopic evaluation following the guidelines of the International Cartilage Repair Society (ICRS) scoring system. After gross examination, the samples were fixed in 10% neutral buffered formalin for approximately 24–48 h, after which the specimens were decalcified in 10% ethylenediaminetetraacetic acid (EDTA) for 30–45 days. Then, the samples were dissected sagittally perpendicular to the surface of the lesion.

The regenerated tissues in paraffin blocks were sectioned into 5-μm sections and stained with HE staining. Cartilaginous matrix distribution was evaluated by safranin O and toluidine blue staining and immunohistochemical staining of collagen type II. The regenerated tissue was graded and analyzed semiquantitatively using a modified ICRS histological scale by 3 independent blinded observers.

#### High-throughput transcriptome analysis

Equal amounts of RNA were isolated from radial shockwave-treated SCB-SPCs and the untreated group using TRIzol reagent. High-throughput transcriptome analysis was performed by Genewiz (Suzhou, China). Briefly, after base calling the original sequences by Bcl2fastq (v2.17.1.14), quality control was conducted by FastQC (v0.10.1). Then, the original data were filtered by Cutadapt (version 1.9.1). Clean data were compared with the reference genome using Hisat2 (v2.0.1). New transcript prediction was performed using StringTie (v1.3.3b) and Cuffcompare (v2.2.1). SNV and InDel analyses were conducted using samtools (v0.1.19) and annovar (v2013.02.11). Gene expression analysis was conducted using Htseq (v.0.6.1). Differentially expressed genes were screened by threshold values of ≥ 2-fold change and *P* value ≤ 0.05. Gene Ontology enrichment analysis was performed according to the categories molecular function, cellular component, and biological process. KEGG (Kyoto Encyclopedia of Genes and Genomes) analysis was also performed. The bioinformatics analyses of differentially expressed genes were performed following the manufacturers’ instructions. The genes correlated with cell proliferation or self-renewal and mechanotransduction-associated components such as the cytoskeleton were screened out and selectively validated by RT-PCR.

### Statistical analysis

All data are presented as the mean values with standard deviations. Independent t test or one-way analysis of variance (ANOVA) followed by post hoc comparisons were employed for normally distributed quantitative data. *P* < 0.05 was considered statistically significant. All tests were analyzed using IBM SPSS Version 20.0.

## Results

### Stem/progenitor cells remain present in human subchondral bones

**Fig. 1:**
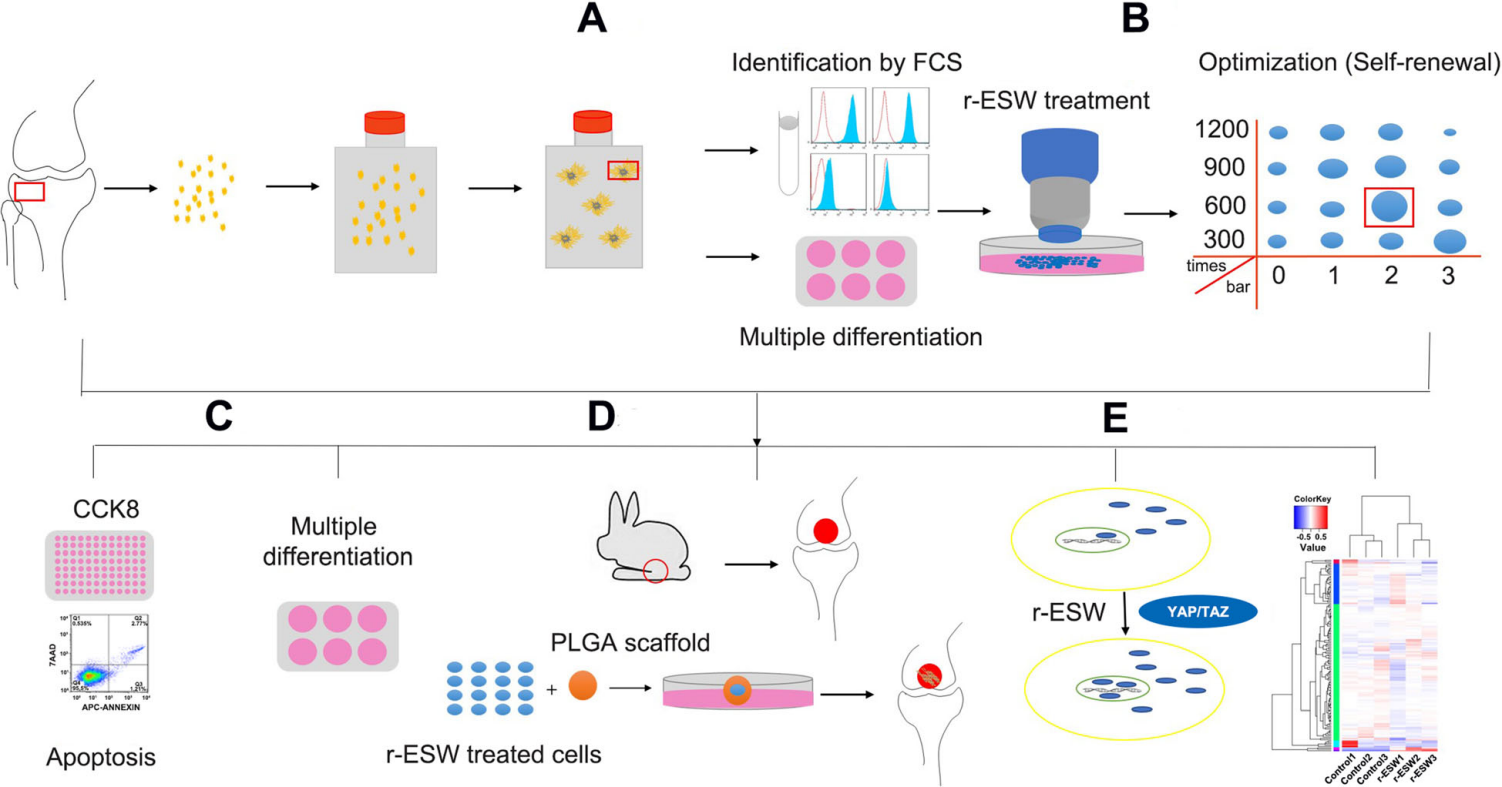
Isolation, cultivation and characterization of SCB-SPCs. **a** Representative images showing the protocols. The marked subchondral bone region of the lateral tibia plateau was cut into bone pieces, rinsed, mildly digested, and then incubated in a culture flask. SCB-SPCs outgrew from bone chips after approximately 10 days of cultivation. The P0 cells were passaged successively to P3 (*n* = 10 donors). **b** Stem/progenitor cell surface immunophenotype expression (CD29, CD31, CD44, CD45, CD73, CD90, CD105, CD166, and CD271) was identified via flow cytometric analysis (*n* = 5). **c** The multiple differentiation potency of SCB-SPCs was examined by induction assay and staining. HE, safranin O, toluidine blue staining, and collagen II and Sox9 immunohistochemical staining to detect chondrogenesis after micropellet culture. ALP staining for osteogenesis. Oil Red O staining for adipogenesis. Scale bars represent 500 μm (ALP staining), 100 μm (Oil Red O staining), and 200 μm (chondrogenic-related staining). SCB-SPCs, subchondral bone stem/progenitor cells

**Fig. 2:**
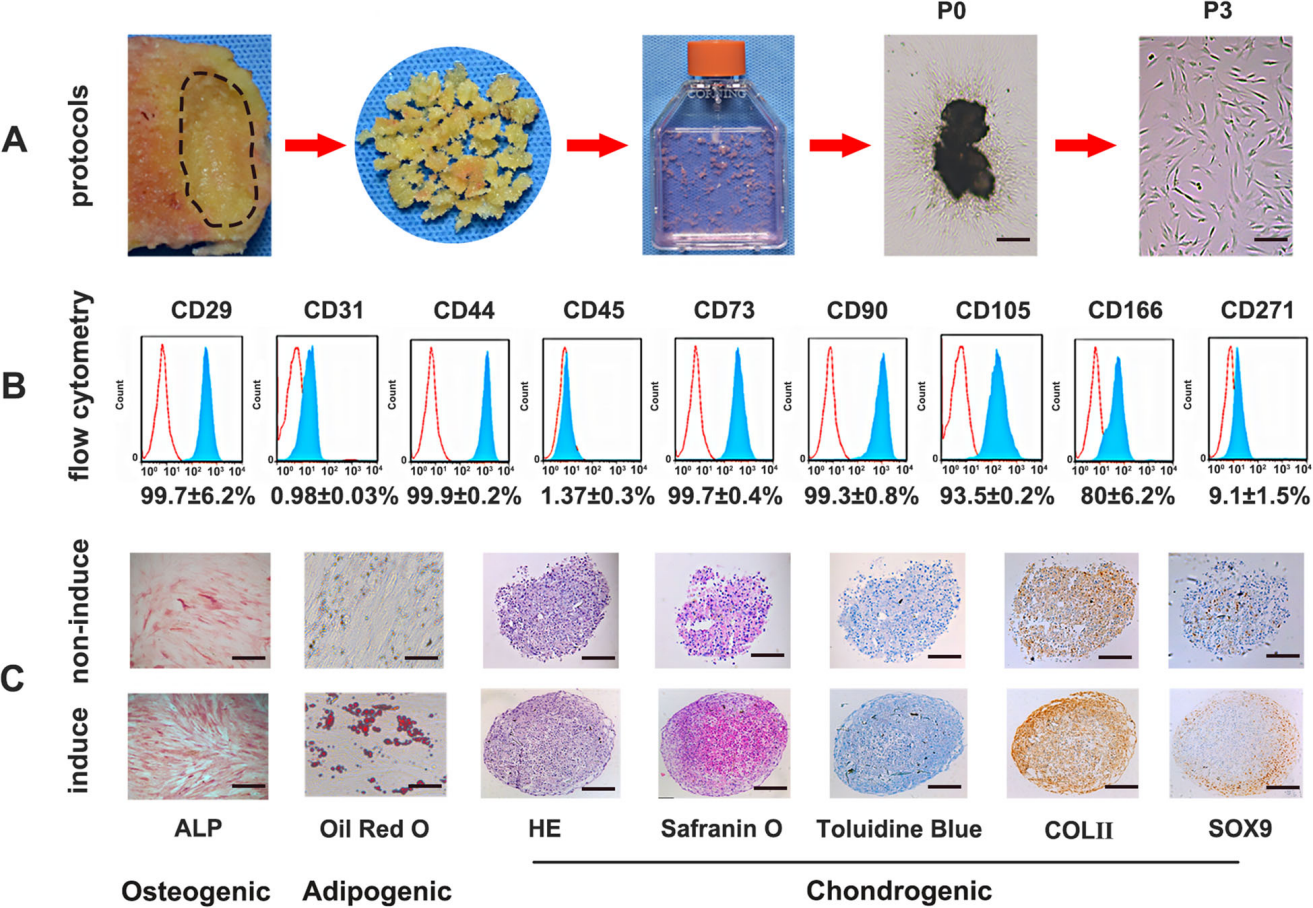
General flow diagram of the experiment. **a** Isolation, cultivation, and characterization of SCB-SPCs. **b** Optimization of r-ESW parameters for the self-renewal of SCB-SPCs. **c** The influences of the optimized parameters on the proliferation, apoptosis, and multidifferentiation of SCB-SPCs. **d** The in vivo therapeutic effects of SCB-SPCs pretreated by r-ESW via an osteochondral lesion model. **e** The underlying mechanisms involved in the mechanotransduction process mediated by r-ESW were explored. SCB-SPCs, subchondral bone stem/progenitor cells. r-ESW, radial extracorporeal shockwave

The isolation protocols were based on previous studies with minor modifications [27]. After approximately 7–10 days of initial culture, fibroblast-like cells could be observed migrating around the subchondral bone chips, and the cells exhibited an adherent growth state. As time increased, an increasing number of cells migrated from the chips (Figs. 1a, and 2a). Flow cytometry analysis revealed that SCB-SPCs were positive for the expression of mesenchymal stem cells with regard to CD29 (99.7 ± 6.2%), CD44 (99.9 ± 0.2%), CD73 (99.7 ± 0.4%), CD166 (80 ± 6.2%), and the stemness-related markers CD90 (99.3 ± 0.8%) and CD105 (93.5 ± 0.2%). Meanwhile, SCB-SPCs were negative for the expression of the endothelial or hematopoietic markers CD31 (0.98 ± 0.03%) and CD45 (1.37 ± 0.3%), further supporting the characteristics of stem/progenitor cells (Fig. 2b).

Regarding multipotency, ALP activity, concomitant with significant increased expression levels of osteogenic markers Runx2 and osteocalcin after inducing culture reveal the osteogenic capacity of SCB-SPCs. The SCB-SPCs also exhibited visible intracytoplasmic lipid droplet accumulation through Oil Red O staining, associated with corresponding elevated gene expression levels of adipogenic markers such as CEBPα and PPARγ. The chondrogenic capacity was evaluated by HE, toluidine blue, and safranin O staining and immunohistochemical staining of Sox9 and collagen II after culturing in a three-dimensional pellet media system. The induced group showed spacious cartilage matrix and significant increases in cartilage-associated markers, including proteoglycan, aggrecan, collagen II, and Sox9 (Fig. 2c, Fig. S2).

### r-ESW promoted self-renewal of SCB-SPCs in a dose-dependent manner

**Fig. 3:**
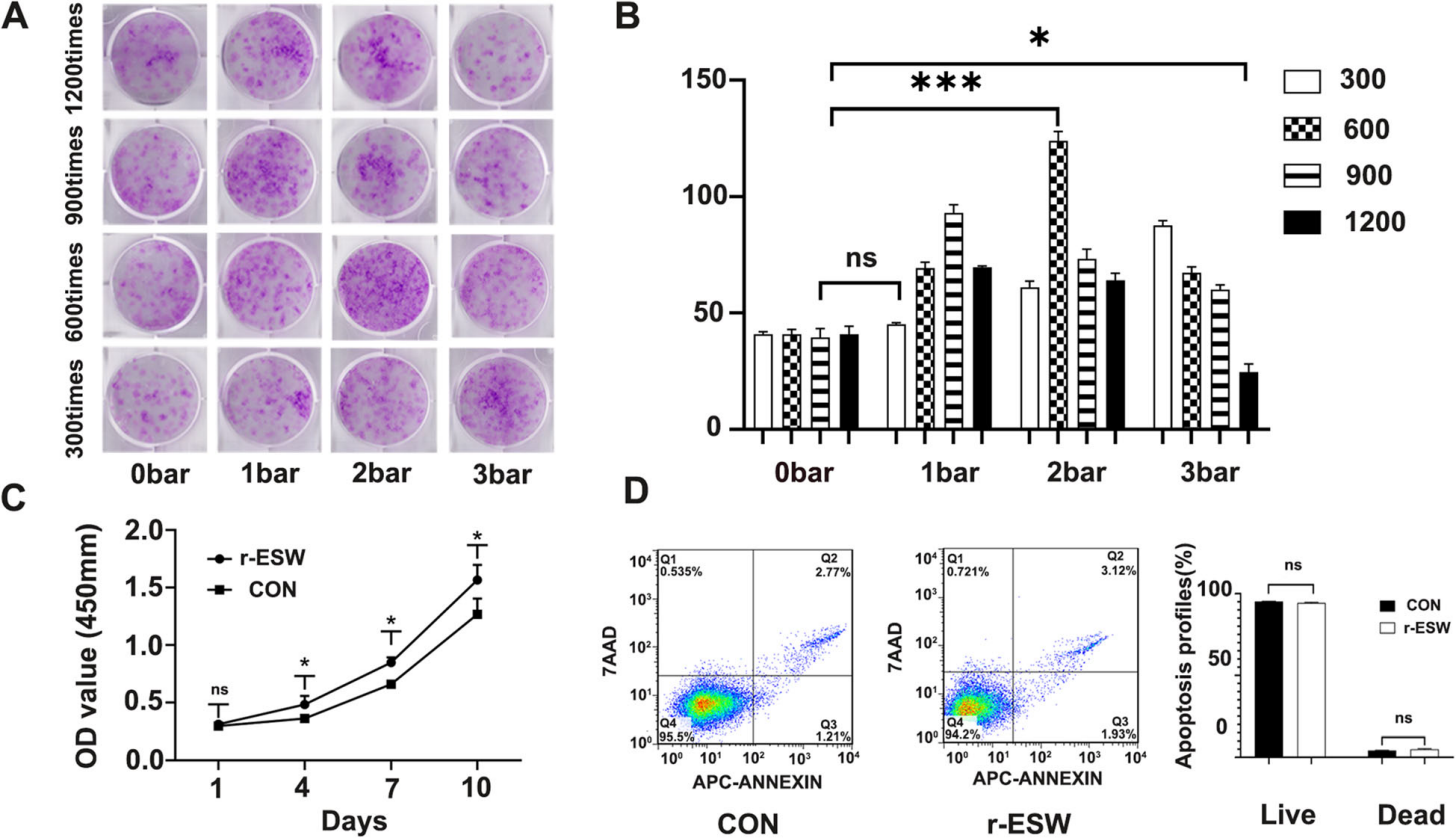
Optimization of radial shockwave parameters. **a** Representative images of CFU-F stained by crystal violet after gradient doses of radial shockwave stimulation. **b** Quantitative comparison of colony numbers in each group based on CFU-F results. The combined analysis suggests that radial shockwave promotes the self-renewal of SCB-SPCs in a dose-dependent manner (*n* = 5, with each 3 technical repeats). **c** The optimized parameters of radial shockwave significantly enhance the proliferation of SCB-SPCs (*n* = 5, with each 3 technical repeats). **d** Compared with the control group, the optimized radial shockwave stimulation had no significant influence on the apoptosis of SCB-SPCs (*n* = 5, with each 3 technical repeats). *p < 0.05, ***p < 0.001, ns, not significant. SCB-SPCs, subchondral bone stem/progenitor cells

A colony-forming unit fibroblast (CFU-F) formation assay was performed after the SCB-SPCs were pretreated with gradient doses of radial shockwave in the floating system (Fig. 1b). The crystal violet staining results showed that the radial shockwave-treated group exhibited larger and denser colonies than the untreated group. However, the positive effects may decrease and even reverse to negative when the energy exceeds a certain range. According to the staining and qualitative analysis results of colonies under microscopy, we found that when the pressure was 1 bar, with the increase in stimulation number from 300 to 900 times, self-renewal exhibited a growing trend, after which self-renewal tended to decrease. At 2 bar, the progressive increase effects of r-ESW only existed from 300 to 600 stimulations. Similarly, when the pressure was 3 bar, as the treatment numbers increased, the self-renewal gradually decreased. The optimal parameter (2 bar, 600 times) was screened out for the following experiments (Fig. 3a, b).

The CCK8 results showed that compared to the control group, the r-ESW group significantly accelerated the proliferation of SCB-SPCs (Fig. 3c). Meanwhile, r-ESW treatment exerted no significant influence on apoptosis (Fig. 3d).

### r-ESW differentially influences tridifferentiation of SCB-SPCs

**Fig. 4:**
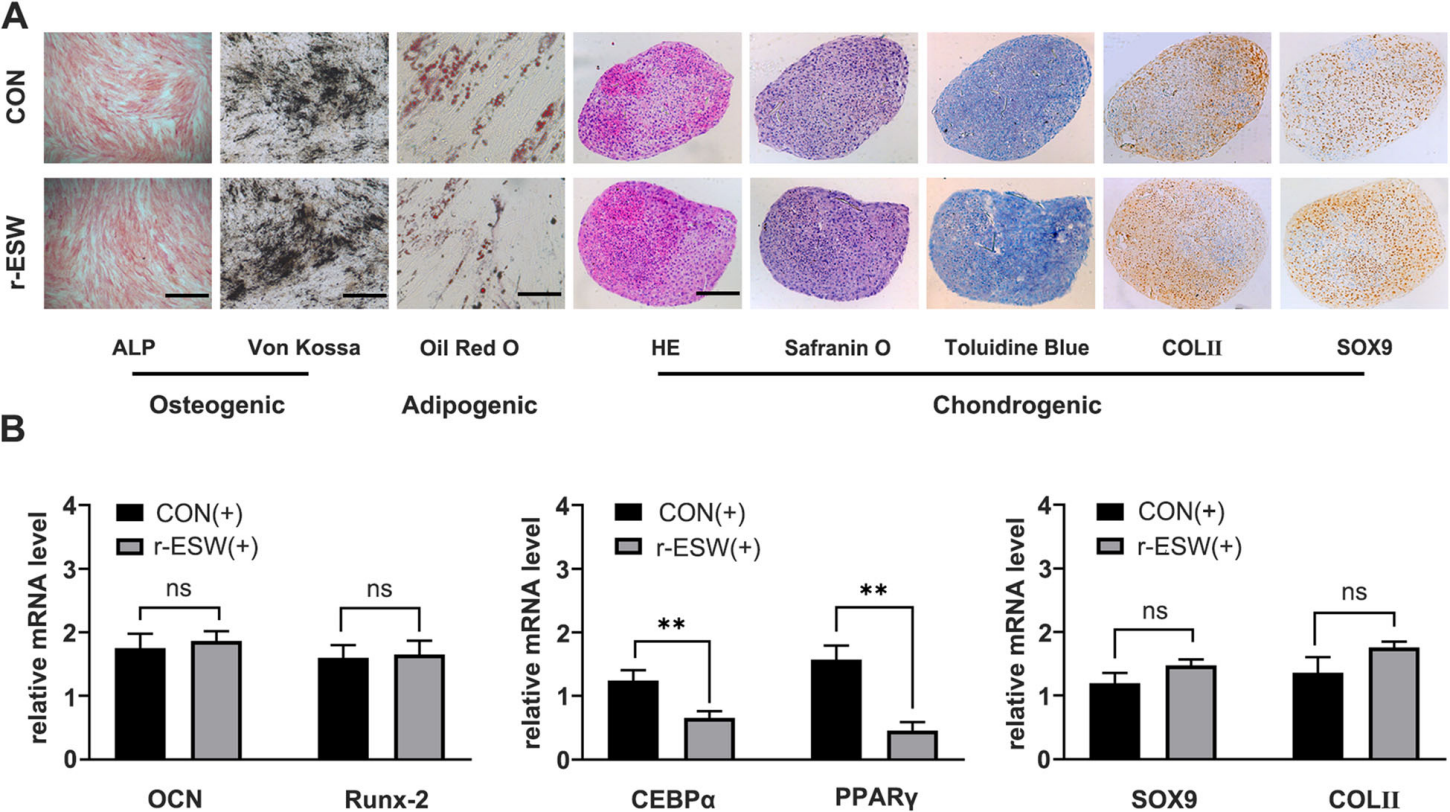
The influences of radial shockwave on the multidifferentiation of SCB-SPCs. **a** ALP and Von Kossa staining indicated that radial shockwave had no significant influence on the osteogenic capacity of SCB-SPCs. Oil Red O staining showed that few positive intracytoplasmic lipid droplets accumulated in radial shockwave-treated SCB-SPCs, and adipogenesis was suppressed by radial shockwave. For chondrogenic potentiality, the treated SCB-SPCs mildly increased, while the change was not significant (*n* = 5, with each 3 technical repeats). Scale bars represent 500 μm (ALP and von Kossa staining), 100 μm (Oil Red O staining), and 200 μm (chondrogenic-related staining). **b** The influences of r-ESW on SCB-SPC tridifferentiation were further validated by the mRNA expression level. Runx2 and OCN for osteogenesis, CEBPα and PPARγ for adipogenesis, Sox9 and Col II for chondrogenesis (*n* = 5, with each 3 technical repeats). The results were consistent with the staining outcomes. *p < 0.05, **p < 0.01, ns, not significant

To further explore the influence of optimized radial shockwave stimulation on SCB-SPC multipotency, induction experiments were performed. In terms of osteogenic potential, no significant difference was detected concerning ALP activity and mineralized matrix accumulation between the r-ESW group and the nontreated group. Consistent with the staining results, the mRNA expression levels of osteogenic genes, including Runx2 and OCN, also revealed no significant differences between the two groups. Regarding adipogenic potential, few Oil-Red-O-positive intracytoplasmic lipid droplet accumulations were discovered in radial shockwave-treated SCB-SPCs, while more lipid droplets formed in the control group. The mRNA expression levels of CEBPα and PPARγ further support the above staining outcomes. In terms of chondrogenic potency, the radial shockwave-treated group exhibited richer aggrecan accumulation in the extracellular matrix through safranin O and toluidine blue staining. Moreover, collagen type II and Sox9 were more abundant in the experimental group. The mRNA expression levels of Sox9 and Col II also increased slightly. However, the change was not significant (Fig. 4).

### r-ESW pretreatment markedly enhances SCB-SPC-mediated cartilage repair in vivo

**Fig. 5:**
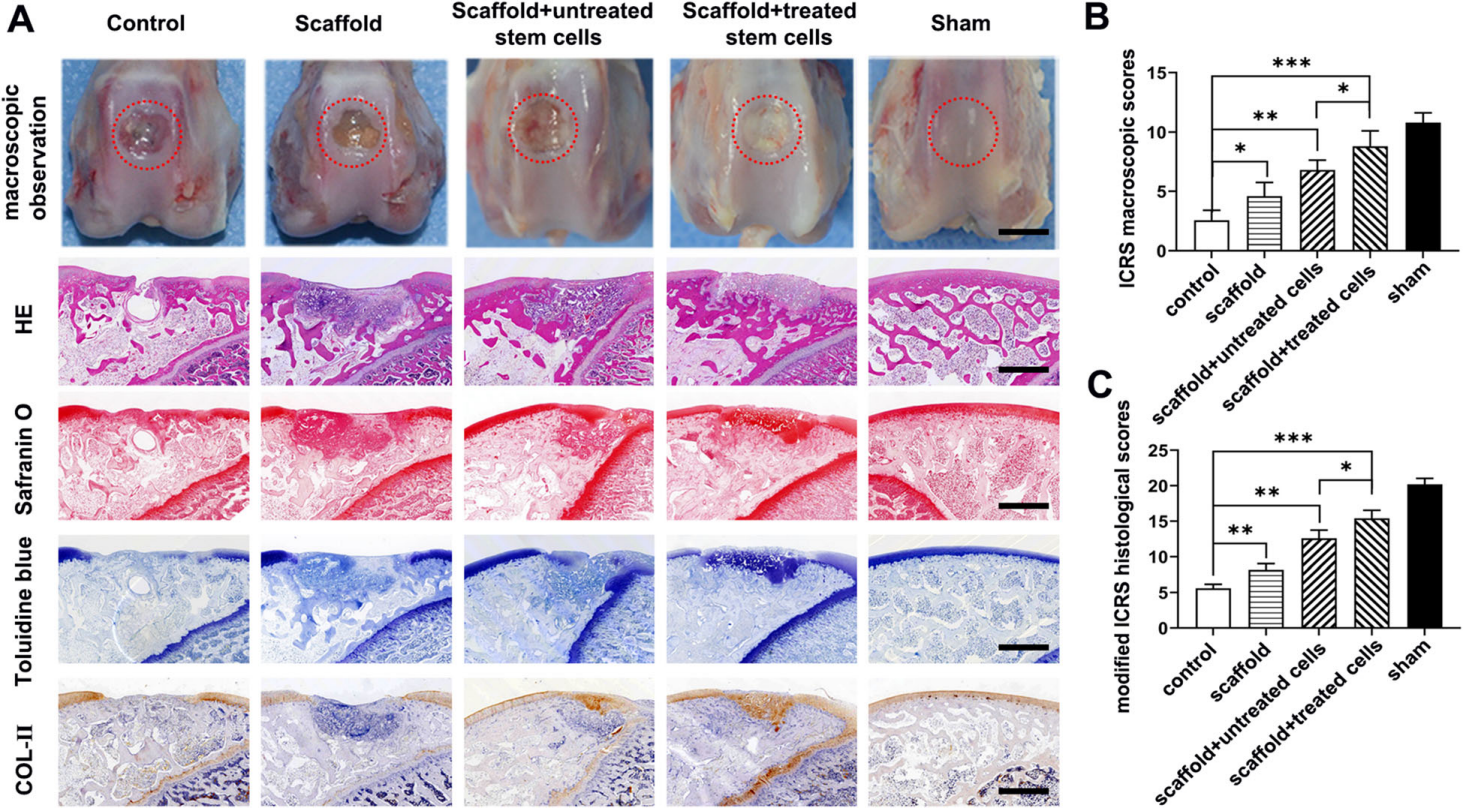
Macroscopic and histologic assessment of cartilage repair effects of SCB-SPCs pretreated by radial shockwave in vivo at 6 weeks. **a** Macroscopic observation of cartilage repair in the femoral trochlea at 6 weeks after surgery. Representative HE, safranin O, toluidine blue, and immunohistochemical staining of Col II. Scale bars represent 1 mm. **b** ICRS rating scores of the regenerated tissue in each group (*n* = 5). **c** Modified ICRS histological scores of cartilage repair in each group (*n* = 5). *p < 0.05, **p < 0.01, ***p < 0.001

Trochlear osteochondral samples were harvested at 6 and 12 weeks after surgery for macroscopic and histologic evaluation. No infection was discovered in any rabbits. At 6 weeks, only a few visually visible fibrous films filled the bottom of the osteochondral defects, and the defects remained clear in the model group. In the scaffold only group, approximately 80% of the osteochondral defect area was filled with fibrous-like repair tissue. When the PLGA scaffold was loaded with untreated SCB-SPCs, newly formed tissue covered approximately 90% of the defect area; however, the neo-tissue was bumpy and irregular. The osteochondral defects in the PLGA scaffold loaded with the radial shockwave-primed SCB-SPCs group were nearly covered with newly formed tissue that had a relatively smooth surface and obscure demarcation from surrounding cartilage (Fig. 5a).

**Fig. 6:**
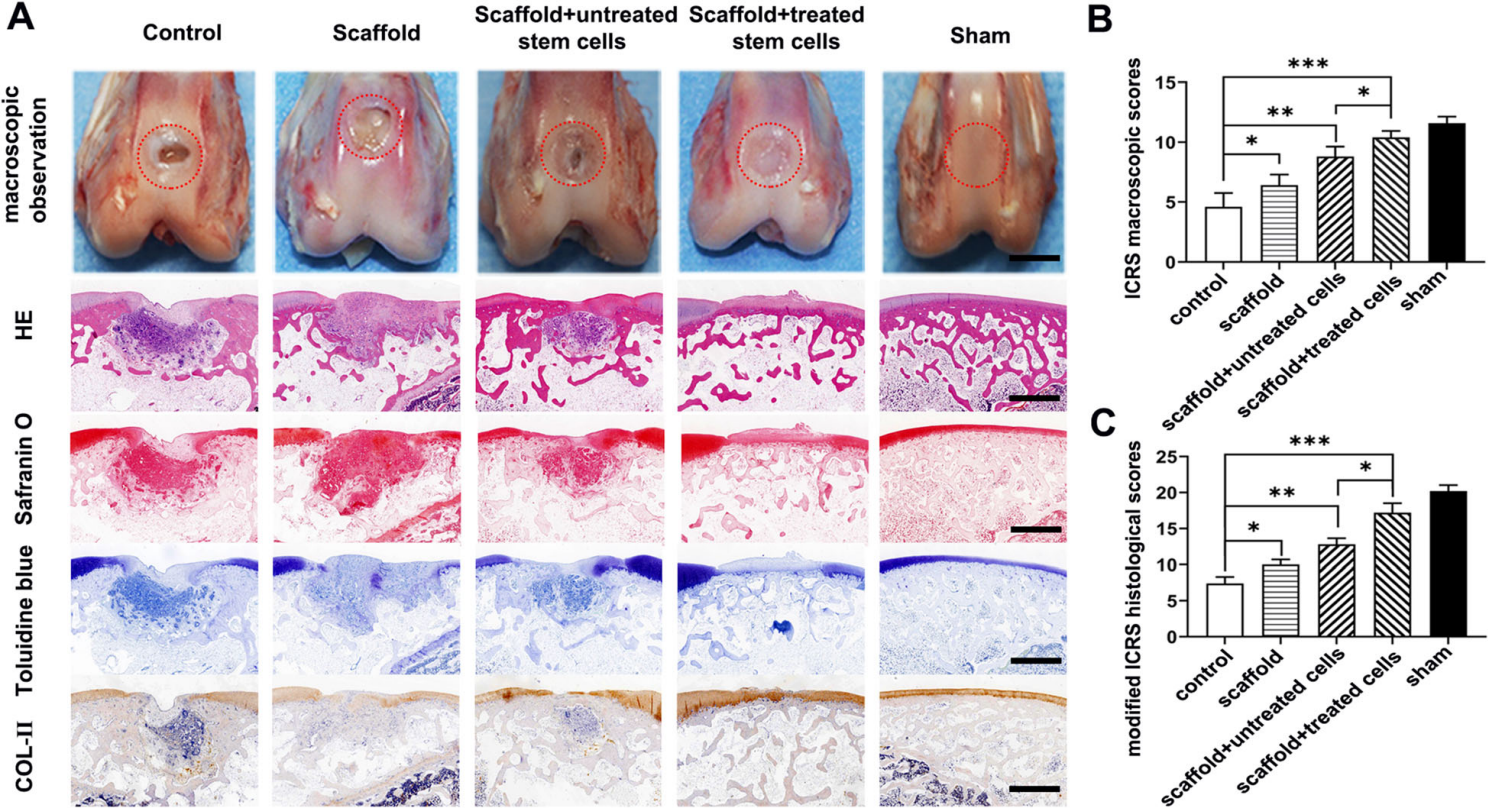
Macroscopic and histologic assessment of cartilage repair effects of SCB-SPCs pretreated by radial shockwave in vivo at 12 weeks. **a** Macroscopic observation of cartilage repair in the femoral trochlea at 12 weeks after surgery. Representative HE, safranin O, toluidine blue, and immunohistochemical staining of Col II. Scale bars represent 1 mm. **b** ICRS scores of the regenerated tissue in each group (*n* = 5). **c** Modified ICRS histological scores of cartilage repair in each group (*n* = 5). *p < 0.05, **p < 0.01, ***p < 0.001

Macroscopic evaluation revealed that in all groups, the repair rate of defect depth, demarcating border, and surface smoothness increased with time. At 12 weeks, the regenerated tissue in the control group was glossy white, indicating the formation of fibrous cartilage; however, the repair area was largely limited to the periphery of the lesion, and there was still a significant defect in the center. In the scaffold only group, white fibrous-like repair tissue covered approximately 90% of the defect area; however, there was still an obvious line between the regenerated and native tissue. The osteochondral defects in the scaffold combined with SCB-SPCs were almost 80% covered with hyaline cartilage-like tissue and were filled with obscure demarcation from surrounding cartilage. Encouragingly, in the scaffold- and radial shockwave-pretreated SCB-SPC groups, the regenerated tissues were all glossy white and integrated with the surrounding normal cartilage, and the surface was smoother and phenotypically much closer to normal cartilage (Fig. 6a). Relative quantitative evaluation based on the ICRS scoring system further supports gross observation. The ICRS score was highest in the scaffold- and radial shockwave-pretreated SCB-SPC groups (Fig. 5b, Fig. 6b).

HE staining was implemented to identify the general characteristics of the repaired tissues. Toluidine blue, safranin O, and collagen type II stainings were employed to investigate the cartilage matrix accumulation level. At 6 weeks after implantation, both images showed distinct borders between regenerated and surrounding tissue, and the neocartilage matrix was shallow compared with the native tissue in all groups. At 12 weeks, the defects remained concave, although with a smoother surface. The pathological analysis discriminated the difference in the repair effects between individual groups and further validated the macroscopic evaluation results.

HE staining of the untreated group showed that the osteochondral defect remained concave with only a few fibrous tissue and inflammatory cells at the bottom and periphery. Toluidine blue and safranin O staining and immunochemistry of Col II showed few positive staining areas. In the scaffold group, the regenerated tissue was larger than that in the control group; however, most of the reparative tissues were nonfunctional fibrous scars with a border between repair tissue and normal cartilage. Special and immunological staining further complemented the above results with sporadically positive histochemical staining. In contrast, the defects were filled with hyaline-like cartilage with PLGA scaffold/SCB-SPC constructs. More importantly, the scaffold/SCB-SPCs pretreated with r-ESW exhibited a more desirable effect with good integration into normal cartilage, although the content of cartilaginous matrix was slightly less abundant than that of normal cartilage (Fig. 6a). Modified ICRS pathological scores further validated the above results (Fig. 6c).

### YAP activation and nuclear translocation contributed to r-ESW-mediated self-renewal enhancement of SCB-SPCs

**Fig. 7:**
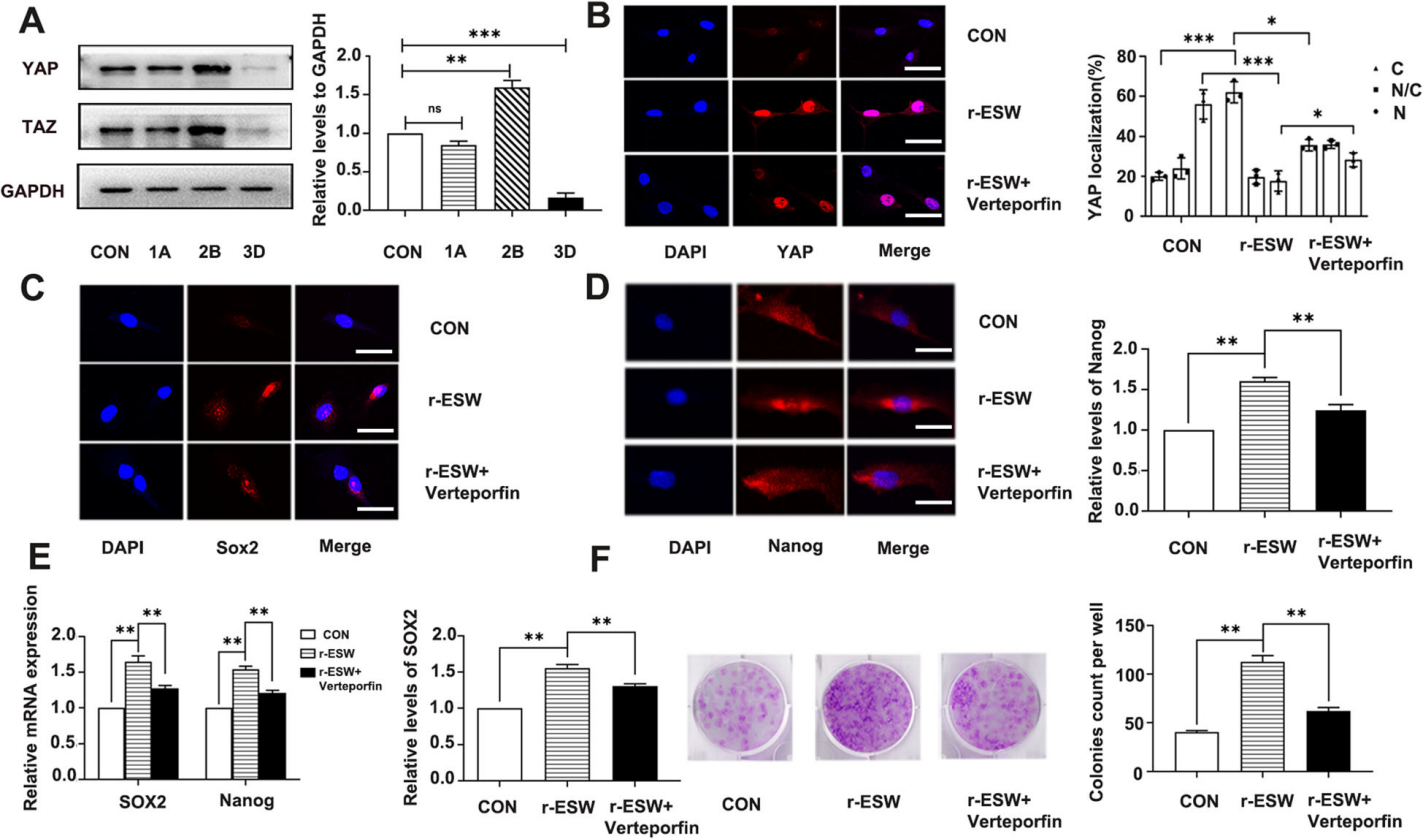
YAP activation and nuclear translocation contributed to radial shockwave-mediated promotion of self-renewal of SCB-SPCs. **a** The YAP/TAZ expression level exhibited a consistent tendency with CFU-F under the respective doses of r-ESW stimulation: control group, 1A (1 bar, 300 times), 2B (2 bar, 600 times), and 3D (3 bar, 1200 times) (*n* = 5, with each 3 technical repeats). **b** Immunofluorescence of YAP localization and semiqualitative evaluation of YAP distribution in SCB-SPCs among the control, r-ESW, and r-ESW + verteporfin groups (*n* = 5, with each 3 technical repeats). **c** Immunofluorescence of Sox2 expression and semiqualitative evaluation in SCB-SPCs among the control, r-ESW, and r-ESW + verteporfin groups (*n* = 5, with each 3 technical repeats). **d** Immunofluorescence of Nanog expression and semiqualitative evaluation in SCB-SPCs among the control, r-ESW, and r-ESW + verteporfin groups (*n* = 5, with each 3 technical repeats). **e** The relative expression levels of self-renewal-associated genes in SCB-SPCs among the control, r-ESW, and r-ESW + verteporfin groups (*n* = 5, with each 3 technical repeats). **f** The general staining and qualitative analysis of CFU-F among the control, r-ESW, and r-ESW + verteporfin groups (*n* = 5, with each 3 technical repeats). Scale bars represent 25 μm. *p < 0.05, **p < 0.01, ***p < 0.001

To explore the possible mechanisms of radial-shockwave potentiated self-renewal, three gradient doses of radial-shockwave on the basis of CFU-F evaluation outcomes were selected (Fig. 3a): a blank control group, a 1A group (1 bar, 300 times), a 2B group (2 bar, 600 times), and a 3D group (3 bar, 1200 times). The protein samples were collected after radial shockwave stimulation at the respective doses. The mechanically sensitive effector protein YAP was chosen to explore the potential mechanism. The western blot results showed that the expression level of YAP/TAZ was closely related to CFU-F outcomes. Specifically, compared to the basic expression level of the blank control group, the 1A group had no significant variation, the 2B group had a marked increase, and the 3D group had a conspicuous decline (Fig. 7a). The YAP expression levels in the respective groups showed change tendencies consistent with the corresponding CFU-F phenomenon (Fig. 3a, b). To further confirm the critical role of YAP/TAZ in SCB-SPC self-renewal, the optimal group 2B was selected for subsequent research. The YAP/TAZ pathway-specific inhibitor verteporfin was used to verify the role YAP/TAZ plays in the self-renewal process mediated by radial shockwave. Before this validation, the SCB-SPCs were cultured in medium supplemented with gradient concentrations of verteporfin. Then, the secure dose was selected by microscopic observation and CFU-F evaluation (Supplementary Fig. S1). Compared to the radial-shockwave group, self-renewal of SCB-SPCs significantly decreased in the inhibitor group (Fig. 7f). Further confocal microscopy outcomes suggested that compared with the control group, the YAP nuclear localization proportion significantly increased in the r-ESW group. Moreover, compared with the control group, Nanog and Sox2 contents exhibited consistent tendencies with YAP expression level and status. Importantly, the YAP nuclear location proportion and Nanog and Sox2 expression levels significantly declined in the r-ESW + verteporfin group (Fig. 7b–d). Meanwhile, the mRNA expression levels of the self-renewal-associated genes Nanog and Sox2 exhibited consistent change tendencies with the above results (Fig. 7e).

### Mechanisms besides YAP signaling were involved in r-ESW-mediated regulation of SCB-SPC self-renewal

**Fig. 8:**
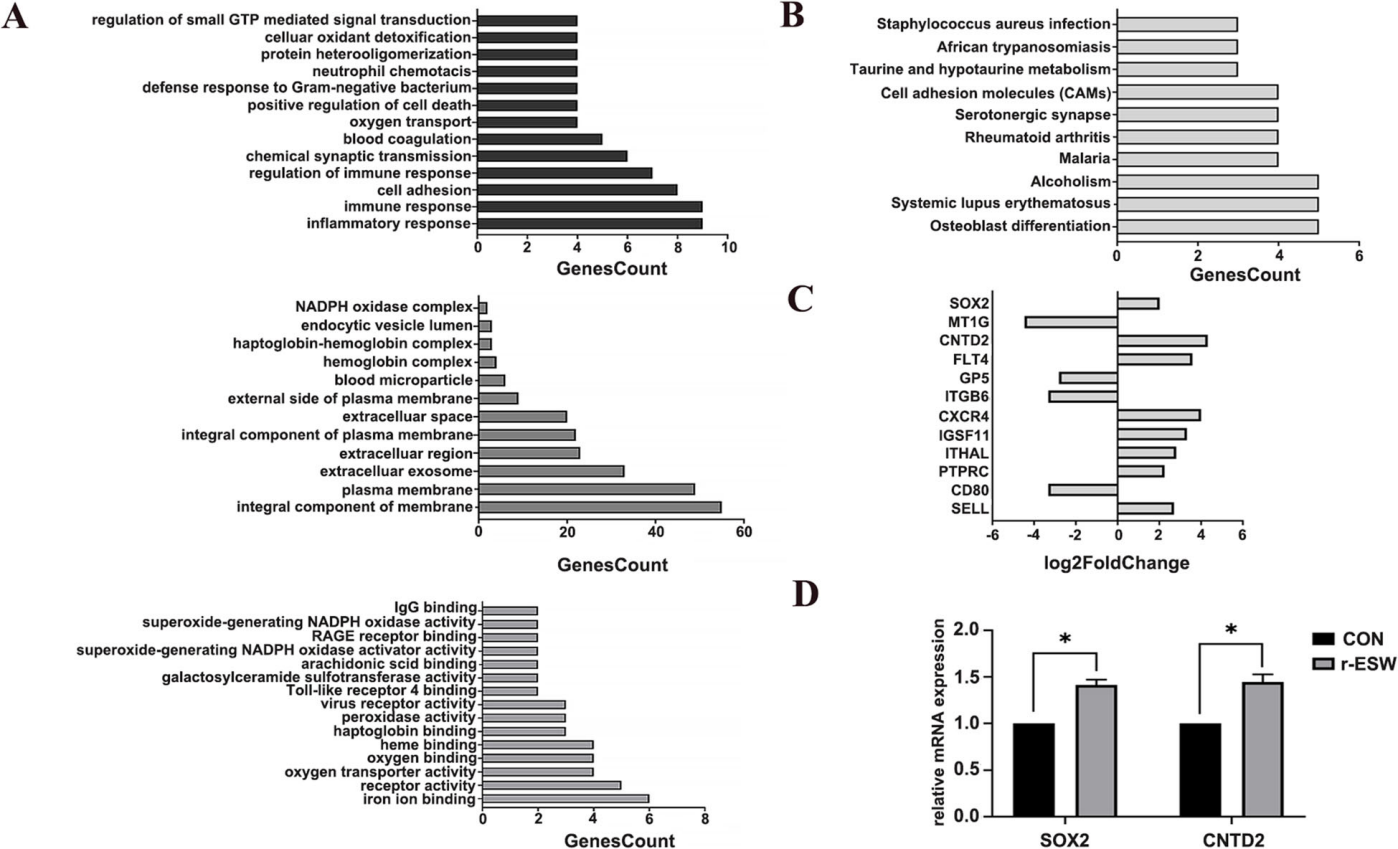
mRNA sequence analysis of SCB-SPCs before and after r-ESW treatment. **a** Classification of differentially expressed genes according to biological process, cell components, and molecular function. **b** KEGG analysis of significantly changed genes after r-ESW treatment. **c** Significantly changed genes related to self-renewal and components that may be involved in the mechanotransduction process are listed. **d** Validation of SOX2 and CNTD2 expression levels by RT-PCR (*n* = 5, with each 3 technical repeats). *p < 0.05

Compared with the untreated group, a total of 600 differentially expressed genes were identified in radial shock wave-treated SCB-SPCs. Three hundred of the genes were upregulated, and 300 were downregulated. Gene ontology (GO) enrichment suggested that the differentially expressed genes were involved in various biological processes, including cell proliferation, adhesion, migration, cytoskeletal transformation, and other biological processes (Fig. 8a, b). Significantly changed genes related to self-renewal, cell adhesion, cell actin cytoskeleton, and ECM-receptor interaction are listed (Fig. 8c). Sox2 and CNTD2 were validated by RT-PCR (Fig. 8d).

## Discussion

In the present study, we demonstrated that radial shockwave is capable of enhancing the self-renewal capacity of SCB-SPCs in vitro, and we screened out the optimized parameters in our experimental system. In addition, primed SCB-SPCs exhibit stronger repair in an osteochondral injury model. Moreover, we identified that the activation of YAP and translocation to the nucleus contributed to this promising effect mediated by radial shockwave.

As a critical factor in regenerative medicine, increasing numbers of tissue-specific stem/progenitor cells have been identified and studied [28–30]. In this study, we chose SCB-SPCs due to their pivotal location and potential therapeutic role in osteochondral repair [13, 31]. Numerous previous studies have indicated the multipotency of SCB-SPCs [19]. Krüger et al. [20] proposed SCB-SPCs as an alternative source for cartilage regeneration. However, the in vivo repair efficiency still needs to be elucidated. Meanwhile, as the repair efficiency correlates closely with the vitality of stem cells, enhancing the tissue-regenerative properties of seed cells is of great significance for clinical therapy. In addition to the commonly used chemical means or gene modifications, radial shockwave and other noninvasive physical means are receiving increasing attention [6, 32–34].

r-ESW has been demonstrated to clinically accelerate the healing of meniscus tears and muscle injuries and has chondroprotective effects [7, 35]. Intriguingly, r-ESW can even strengthen the stemness of stem cells through its unique mechanical characteristics [4, 36]. As one specificity of stem cells, self-replication or self-renewal controls the stem cell pool and determines the repair efficiency. Therefore, in our research, we first optimized the parameters of radial shockwave on SCB-SPCs via CFU-F evaluation. Our results show that radial shockwave influences the self-renewal of SCB-SPCs in a dose-dependent manner. Moreover, we combined shockwave intensity (bar) with stimulation numbers in our protocols. The CFU-F data suggest that the intensity and stimulation times collectively influence the self-renewal of SCB-SPCs. Based on the data, we speculate that a parameter characterizing the total energy may be able to comprehensively represent the compositive effects of intensity and stimulation times. Our results further verify the critical role of radial shockwave energy on biological effects, and the results provide a reference for clinical therapy. Following the optimized parameters based on the CFU-F results, we further explored the biological effect of radial shockwave on multidifferentiation.

Accumulating evidence suggests that shockwave stimulation could regulate the differentiation potential of stem cells [37–39]; however, the effects are still controversial. In the current study, we found that the optimized radial shockwave screened out based on CFU-F has no significant influence on the osteogenic capacity of SCB-SPCs. The outcomes were different from the majority of previous studies in that the osteogenic capacity of mesenchymal stem cells was enhanced after radial shockwave stimulation [37, 38]. This phenomenon may result from the stimulation regimen selected based on CFU-F results, which may not be optimal for differentiation induction. Because the effect of radial shockwave on SCB-SPC differentiation was first reported, the outcomes may still need further validation. Chondrogenic capacity was mildly increased, while the change was not significant. Consistent with our previous work and the literature, the adipogenic potential was significantly suppressed. However, we are aware that different stem cell populations, shockwave stimulation systems, and protocols may contribute to the discrepancy effects. Moreover, protein-based assay would be helpful to further validate our results.

To further investigate the in vivo repair efficiency of shockwave-treated SCB-SPCs, the classical and stable model of osteochondral defects was used. Porous PLGA scaffolds were applied as cell carriers based on our previous study [26]. The gross observations characterized by ICRS scores along with histological analysis following modified ICRS scores of neo-tissues at 2 different points demonstrated that SCB-SPCs pretreated by r-ESW yielded better repair results. The in vivo repair effect validation further strengthens the translational application value of radial shockwave. However, the concrete mechanisms of r-ESW in vivo still need to be explored.

YAP/TAZ, the effector of the Hippo pathway, has been demonstrated to play pivotal roles in mechano-meditated stem cell plasticity and stemness regulation during tissue regeneration [40, 41]. Moreover, numerous studies have suggested that YAP protein activity could be regulated directly by mechanical cues from both the microenvironment, such as extracellular matrix stiffness, and other extraneous stimulation independent of the Hippo pathway [42, 43]. Notably, mechanical stimuli could influence YAP nuclear-cytoplasmic distribution and in turn modulate cell biological function [40, 44]. DuPont et al. identified YAP/TAZ as sensors and effector proteins of mechanical cues perceived from extracellular rigidity [45]. Additionally, YAP/TAZ also responds to different kinds of mechanical stresses, including shearing, compressing, or pulling [40]. Extracorporeal shockwaves, a unique physical stimulus composed of compression, tension, and shear forces, are transient pressure disturbances that propagate rapidly in three-dimensional space. However, whether YAP activity is involved in radial shockwave-mediated biological effects is still unclear. Therefore, we explored its activity in our study. The results demonstrated that the activity of YAP also contributes to the radial shockwave-mediated process of enhancing the self-renewal of SCB-SPCs. To exclude side effects, the YAP-specific inhibitor verteporfin was used to further validate this effect. Previous studies suggested that Sox2 and Nanog are binding targets of YAP/TAZ by genome-wide analysis [46]. We found that with the activation and nuclear translocation of YAP after radial shockwave stimulation, the mRNA and protein expression of Sox2 and Nanog also increased. Importantly, after the YAP-specific inhibitor verteporfin was used, the expression of Sox2 and Nanog significantly decreased. The change tendency of YAP status and the corresponding expression of Sox2 and Nanog are consistent with the self-renewal of SCB-SPCs characterized by CFU-F. The comprehensive analysis of the results suggests that YAP/TAZ and downstream self-renewal-associated genes are involved in the biological process of radial shockwave-mediated promotion of SCB-SPC self-renewal. The results are consistent with previous studies that demonstrated that YAP correlated closely with the self-renewal of ES cells because elevated YAP maintains the potency of ES cells [46, 47]. Meanwhile, others reported that YAP/TAZ are dispensable for ES cell fate [48]. Hence, the cell-specific functional style of YAP/TAZ still needs further exploration. Moreover, the detailed signal activity events between the receptor and ultimate YAP effector still need further investigation. In view of the results that CFU-F in the r-ESW + verteporfin group was stronger than that in the control group, we speculate that other pathways may also be involved in radial shockwave-mediated self-renewal prompting, except the YAP pathway. Hence, high-throughput sequences were implemented. The results revealed that self-renewal-related genes increased after radial shockwave and supported the idea that other self-renewal-associated pathways may also contribute to this effect.

There are still several limitations in the present study. Although a series of gradient parameters have been designed to explore the biological effect of radial shockwave, the quantitative evaluation parameters and corresponding biological effects still need to be explored owing to the complexity of radial shockwaves. In addition, the optimized parameters in the current study were screened out by CFU-F, which may not be optimal for the differentiation capacity of SCB-SPCS. Further precise output of radial shockwave energy will facilitate the optimization of different characteristics of stem cells and accelerate their clinical application. Meanwhile, the SCB-SPCs were cultured in vitro, which may not comprehensively represent the in vivo status. Moreover, the mechanisms of in vivo experiments still need further exploration and validation.

## Conclusion

We found that radial extracorporeal shockwave is capable of promoting the self-renewal of SCB-SPCs in vitro by targeting YAP activity and strengthening its repair efficiency in vivo, indicating promising application prospects.

## Supplementary Information

**Additional file 1: Table S1.** Demographic, clinical, and imaging characteristics of the donors. **Table S2.** Primer sequences for RT-qPCR. **Table S3.** Experiment groups of in vivo study (6 weeks and 12 weeks).

**Additional file 2: Figure S1.** The concentration screening experiment of YAP specific inhibitor verteporfin. (a) primary concentration screening by photomicrographs and CFU-F evaluation under a series of verteporfin concentration; (b) secondary screening on the basis of primary results. The selected concentration was 0.05uM.

**Additional file 3: Figure S2.** The comparison of tridifferentiation associated genes expression level between non-induced and induced group. The relative mRNA expression level of osteogenic related markers (OCN, Runx-2), adipogenic markers (CEBPα and PPARγ), chondrogenic associated markers (Collagen II and Sox9) were significantly higher in induced group. *p < 0.05, **p < 0.01, ***p < 0.001.

**Additional file 4.**

**Additional file 5.**

## Data Availability

The datasets used and/or analyzed during the current study are available from the corresponding author on reasonable request.

## Abbreviations

α-MEM: Alpha-minimal essential medium
CCK8: Cell Counting Kit-8
CEBP/α: CCAAT/enhancer binding protein alpha
CFU-F: Colony-forming unit fibroblast assay
Col-II: Type II collagen
EDTA: Ethylenediaminetetraacetic acid
FBS: Fetal bovine serum
GAPDH: Glyceraldehyde-3-phosphate dehydrogenase
GO: Gene ontology
ICRS: International Cartilage Repair Society
MSCs: Mesenchymal stem cells
OCN: Osteocalcin
K-L: Kellgren-Lawrence
KEGG: Kyoto Encyclopedia of Genes and Genomes
PBS: Phosphate-buffered saline
PI: Propidium iodide
PPARγ: Peroxisome proliferator-activated receptor gamma
PLGA: Polylactic-co-glycolic acid
r-ESW: Radial extracorporeal shockwave
RT-qPCR: Real-time quantitative polymerase chain reaction
Runx-2: Runt-related transcription factor 2
SCB-SPCs: Subchondral bone stem/progenitor cells
Sox-9: Sex determining region Y-box 9
