# Supplementary material for: Radial extracorporeal shockwave promotes subchondral bone stem/progenitor cell self-renewal by activating YAP/TAZ and facilitates cartilage repair in vivo
Source: Stem Cell Res Ther. 2021 Jan 7;12:19. doi: 10.1186/s13287-020-02076-w (PMC7792202; doi:10.1186/s13287-020-02076-w)
Supplement: Supplementary file 1 — Additional file 1: Table S1. Demographic, clinical, and imaging characteristics of the donors. Table S2. Primer sequences for RT-qPCR. Table S3. Experiment groups of in vivo study (6 weeks and 12 weeks). [file 13287_2020_2076_MOESM1_ESM.docx]

**Supplementary Table 1. Demographic, clinical, and imaging characteristics of the donors.**

| ID | Age  (years) | Sex | BMI  (kg/m^2^) | Disease  duration  (years) | K-L grade of  knee lateral compartment |
| --- | --- | --- | --- | --- | --- |
| 1 | 64 | M | 25.5 | 9 | 1-2 |
| 2 | 58 | F | 25.6 | 8 | 1-2 |
| 3 | 70 | F | 26.7 | 11 | 1-2 |
| 4 | 56 | M | 26.4 | 9 | 1-2 |
| 5 | 60 | F | 24.4 | 3 | 1-2 |
| 6 | 61 | M | 25.1 | 4 | 1-2 |
| 7 | 54 | F | 30.8 | 6 | 1-2 |
| 8 | 57 | F | 23.6 | 7 | 1-2 |
| 9 | 58 | F | 27.4 | 4 | 1-2 |
| 10 | 66 | M | 27.3 | 9 | 1-2 |
|  |  |  |  |  |  |

**Table 2. Primer sequences for RT-qPCR**

| genes | primer sequences |
| --- | --- |
| Runx-2 | forward, 5’- CTGTGGTTACTGTCATGGCG-3′  reverse, 5’- CCCTCCCTTTTCCCACTCAT-3′ |
| OCN | forward, 5′- AGGTGCGAAGCCCAGCGGTGCA-3′  reverse, 5′- CCTGGAGAGGAGCAGAACTGGG-3′ |
| CEBP/α | forward, 5′- GGAGGGTCTCTAGTTCCACG-3′  reverse, 5′- CCCACAGCCAGATCTCTAGG-3′ |
| PPARγ | forward, 5′- TTGCAGTGGGGATGTCTCAT-3′  reverse, 5′- TTTCCTGTCAAGATCGCCCT-3′ |
| Sox-9 | forward, 5′- ATGAAGATGACCGACGAGCA -3′  reverse, 5′- AACTTGTCCTCCTCGCTCTC-3′ |
| Col-II | forward, 5′- AGCCTGGTGATGATGGTGAA-3′  reverse, 5′-ACTCTCACCCTTCACACCAG-3′ |
| Nanog | forward, 5′-TGAGTGTGGATCCAGCTTGT-3′  reverse, 5′-TCTCTGCAGAAGTGGGTTGT-3′ |
| Sox-2 | forward, 5′-CATGTCCCAGCACTACCAGA-3′  reverse, 5′-TACCGGGTTTTCTCCATGCT-3′ |
| CNTD2 | forward, 5′-TCTGGCGGTTCACCTGCT-3′  reverse, 5′-TCGGGAAGCACGCACTCT-3′ |
| GAPDH | forward, 5′-GCCATCGTGGCTAAACAGGTA-3′  reverse, 5′-GTTGGTGTTCATCCGCTTGC-3′ |

RT-qPCR: real-time quantitative polymerase chain reaction; Runx-2: runt-related transcription factor 2; OCN: osteocalcin; CEBP/α: CCAAT/enhancer binding protein alpha; PPARγ: peroxisome proliferator-activated receptor gamma; Sox-9: sex determining region Y-box 9; Col-II: type II collagen,

**Table 3. Experiment groups of in vivo study (6 weeks and 12 weeks)**

| Groups | Rabbit number | Treatment means |
| --- | --- | --- |
| Control | 5 | Osteochondral defects without treatment |
| Scaffold | 5 | Osteochondral defects treated with PLGA only |
| Scaffold+ un-treated SCB-SPCs | 5 | Osteochondral defects treated with PLGA +untreated SCB-SPCs |
| Scaffold + treated SCB-SPCs | 5 | Osteochondral defects treated with PLGA+ treated SCB-SPCs |
| Sham | 5 | Sham- |
