# Supplementary figures and images for: Radial extracorporeal shockwave promotes subchondral bone stem/progenitor cell self-renewal by activating YAP/TAZ and facilitates cartilage repair in vivo
Source: Stem Cell Res Ther. 2021 Jan 7;12:19. doi: 10.1186/s13287-020-02076-w (PMC7792202; doi:10.1186/s13287-020-02076-w)

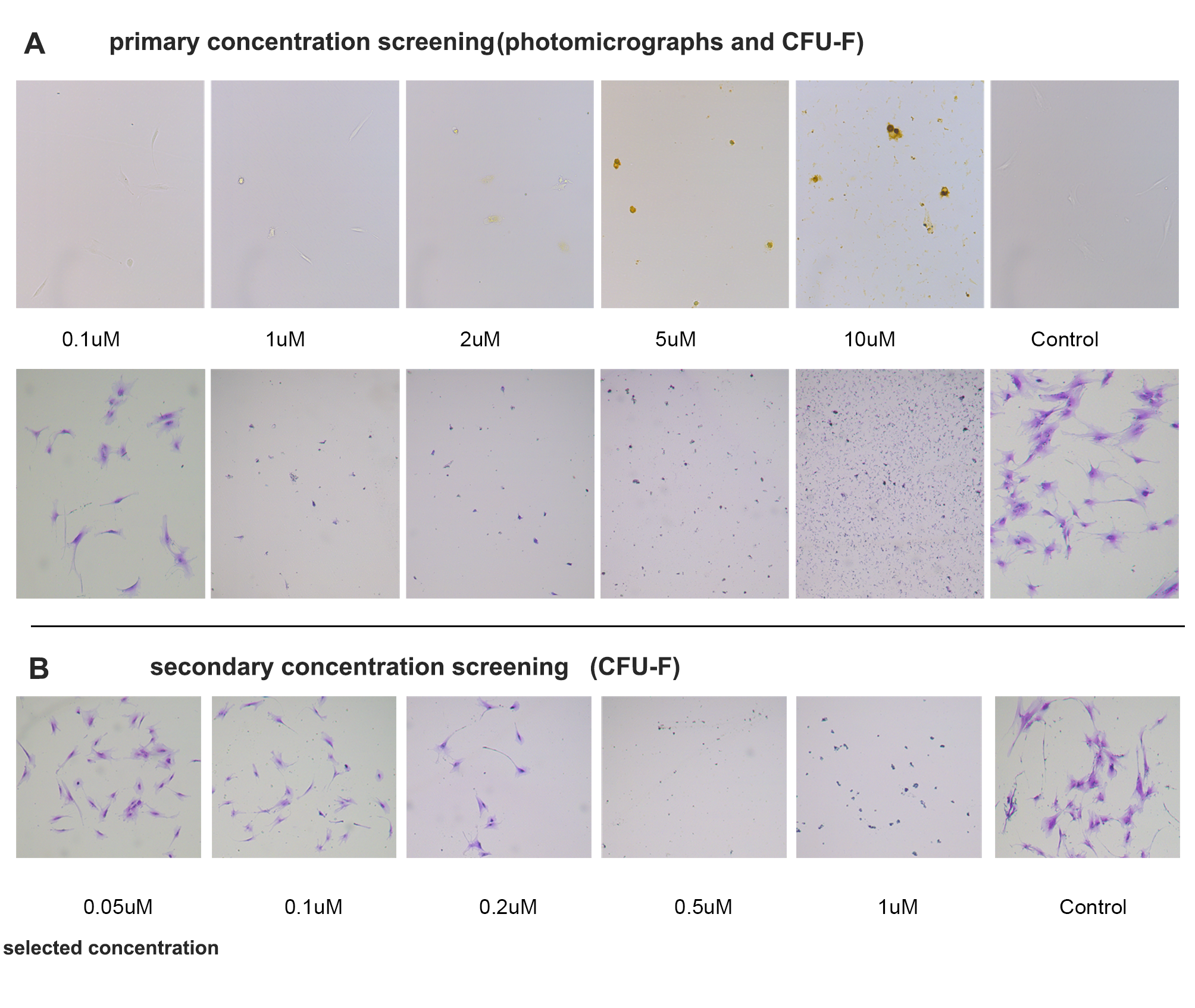

Supplement: Supplementary file 2 — Additional file 2: Figure S1. The concentration screening experiment of YAP specific inhibitor verteporfin. (a) primary concentration screening by photomicrographs and CFU-F evaluation under a series of verteporfin concentration; (b) secondary screening on the basis of primary results. The selected concentration was 0.05uM. [file 13287_2020_2076_MOESM2_ESM.tif]

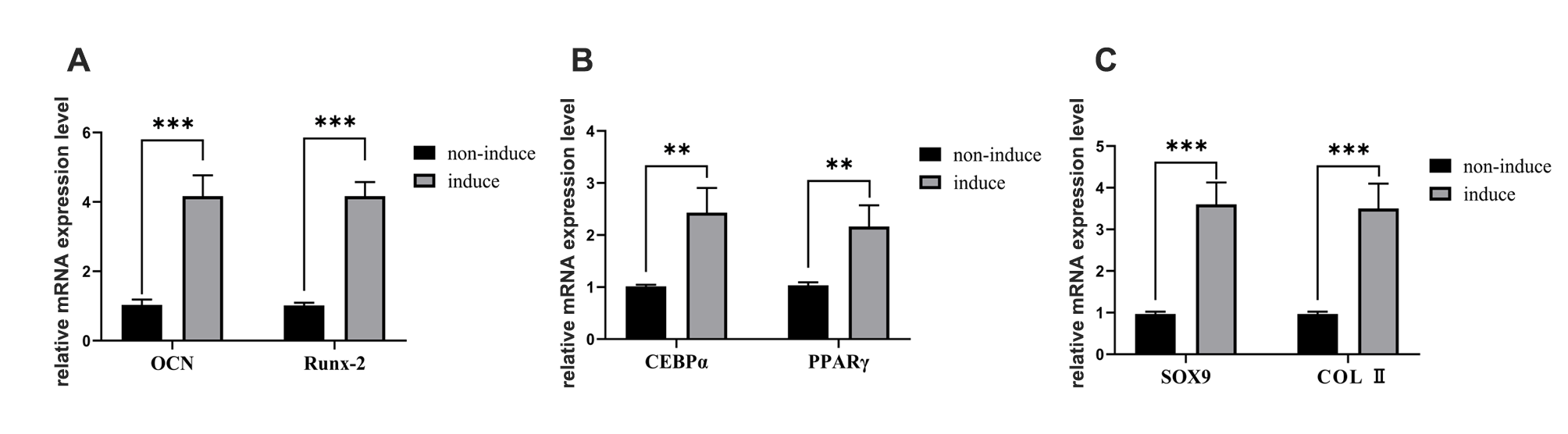

Supplement: Supplementary file 3 — Additional file 3: Figure S2. The comparison of tridifferentiation associated genes expression level between non-induced and induced group. The relative mRNA expression level of osteogenic related markers (OCN, Runx-2), adipogenic markers (CEBPα and PPARγ), chondrogenic associated markers (Collagen II and Sox9) were significantly higher in induced group. *p < 0.05, **p < 0.01, ***p < 0.001. [file 13287_2020_2076_MOESM3_ESM.tif]
