# Supplementary material for: Radial extracorporeal shockwave promotes subchondral bone stem/progenitor cell self-renewal by activating YAP/TAZ and facilitates cartilage repair in vivo
Source: Stem Cell Res Ther. 2021 Jan 7;12:19. doi: 10.1186/s13287-020-02076-w (PMC7792202; doi:10.1186/s13287-020-02076-w)
Supplement: Supplementary file 4 — Additional file 4. [file 13287_2020_2076_MOESM4_ESM.pdf]

解放军总医院伦理审查

科研项目快速审查批件

项目名称	**Discarded biological materials from knee osteoarthritis patients with total knee arthroplasty for scientific research projects**
项目负责人	赵之栋
项目负责人科室	中国人民解放军总医院　外科临床部　骨科
审查文件	研究方案　知情同意书
伦理审查方式	快速审查

依据《药物临床试验质量管理规范》，《涉及人体的生物医学研究伦理审查办法》、《药物临床试验伦理审查工作指导原则》、世界医学会《赫尔辛基宣言》、世界卫生组织《生物医学研究审查伦理委员会操作指南》、国际医学科学组织委员会《涉及人的生物医学研究国际伦理准则》等法律、法规和国际准则，伦理委员会于 2018 年 3 月 15 日对上述试验进行了审阅，认为基本符合伦理要求，可以进行课题申报。

- 本机构伦理委员会批准的项目为涉及人体的生物医学研究，必须严格按照所批最新版本的研究方案和知情同意书开展研究，并遵循国内相关法规指南要求。
- 凡是涉及人类遗传资源出口或者按照国家规定必须经有关部门专项审批的内容，均需在项目执行前向有关部门申报并获得批准。

解放军总医院医学伦理委员会

日期：2018 年 3 月 15 日
