# Supplementary material for: Radial extracorporeal shockwave promotes subchondral bone stem/progenitor cell self-renewal by activating YAP/TAZ and facilitates cartilage repair in vivo
Source: Stem Cell Res Ther. 2021 Jan 7;12:19. doi: 10.1186/s13287-020-02076-w (PMC7792202; doi:10.1186/s13287-020-02076-w)
Supplement: Supplementary file 5 — Additional file 5. [file 13287_2020_2076_MOESM5_ESM.pdf]

解放军总医院实验动物福利伦理审查批件

编号：2019-X15-57　　　　　　　　　　　　签署日期：2019-04-13

申请题目	精准能量冲击波修复软骨损伤的实验研究		
申 请 人	赵之栋	申请日期	2019-04-09
申请单位	中国人民解放军总医院		
联系电话	18703416131	申请编号	2019-X15-57
审查意见	通过审查（ ✓ ）		
	修改后通过（修改意见：）		
	未通过审查（不同意理由：）		

签字页

姓 名	人员类别*	签 名
夏 蕾	科研管理	
何昆仑	科研管理	
刘广东	科研管理	
颜光涛	医学研究	
翟所强	医学研究	
崔俊昌	医学研究	
郭明洲	医学研究	
白雪源	医学研究	
张爱群	医学研究	
张 余	公众代表	
主任委员意见	签名: [illegible]	日期: 2019. 4.13
